# Pterostilbene and 4′-Methoxyresveratrol Inhibited Lipopolysaccharide-Induced Inflammatory Response in RAW264.7 Macrophages

**DOI:** 10.3390/molecules23051148

**Published:** 2018-05-11

**Authors:** Yun Yao, Kehai Liu, Yueliang Zhao, Xiaoqian Hu, Mingfu Wang

**Affiliations:** 1 College of Food Science and Technology, Shanghai Ocean University, No.999 Hu-Cheng-Huan Road, Shanghai 201306, China; yaoyun0111@163.com (Y.Y.); khliu@shou.edu.cn (K.L.); mfwang@hku.hk (M.W.); 2 Laboratory of Quality and Safety Risk Assessment for Aquatic Products on Storage and Preservation (Shanghai), Ministry of Agriculture, Shanghai 201306, China

**Keywords:** pterostilbene, 4′-methoxyresveratrol, inflammation, NF-κB, MAPK, AP-1

## Abstract

Pterostilbene (Pte) and 4′-Methoxyresveratrol (4MR) are methylated derivatives of resveratrol. We investigated the anti-inflammatory effect of Pte and 4MR in lipopolysaccharide (LPS)-stimulated RAW264.7 murine macrophages. Both Pte and 4MR significantly reduced LPS-induced nitric oxide release by inhibiting the inducible nitric oxide synthase mRNA expression. Moreover, both of them inhibited LPS-induced mRNA expression of inflammatory cytokines including monocyte chemoattractant protein (MCP)-1, interleukin (IL)-6 and IL-1β, and tumor necrosis factor α (TNF-α), and attenuated LPS-induced nuclear factor-κB (NF-κB) activation by decreasing p65 phosphorylation. In addition, 4MR but not Pte inhibited LPS-induced the activator protein (AP)-1 pathway in RAW 264.7 macrophages. Further study suggested that Pte had an inhibitory effect on extracellular regulated protein kinases (ERK) and p38 activation, but not on c-Jun N-terminal kinase (JNK), while 4MR had an inhibitory effect on JNK and p38 activation, but not on ERK. Taken together, our data suggested that Pte induced anti-inflammatory activity by blocking mitogen-activated protein kinase (MAPK) and NF-κB signaling pathways, while 4MR showed anti-inflammatory activity through suppression of MAPK, AP-1, and NF-κB signaling pathways in LPS-treated RAW 264.7 macrophages.

## 1. Introduction

Inflammation is a kind of body defensive response to external stimuli. It can destroy and remove the detrimental agents and injured tissues, thereby benefiting tissue repair [1]. However, when this protective response is out of control, excessive cell and tissue damage will occur and lead to many diseases [2], such as diabetes [3], colitis [4], rheumatism [5], and atherosclerosis [6]. Thus, anti-inflammatory agents may be therapeutically useful.

Macrophages play an essential role in the inflammatory response. The RAW264.7 cell line is well accepted as a suitable macrophage model [7]. Lipopolysaccharide (LPS) is commonly used for the induction of inflammatory models for its ability to regulate the expression of inflammatory-related enzymes and release of inflammatory mediators by activating multiple signaling pathways [8]. In LPS-induced RAW264.7 cells, toll-like receptor 4 (TLR4) recognizes and binds to LPS, promoting the association of TLR4 with the adaptor myeloid differentiation factor 88. Subsequently, progressive inflammatory signaling induces activation of mitogen-activated protein kinases (MAPKs), AP-1, and NF-κB protein kinases, eventually contributing to the inflammatory response [9,10].

AP-1 is a crucial transcription factor regulating inflammation response [11]. LPS activates AP-1 proteins (Fos and Jun heterodimers), which generally regulate their transcriptional activity through interactions with adjacent proteins or with transcriptional coactivators at active domains to regulate the generation of inflammatory cytokines such as TNF-α, IL-1β, and IL-6 [12,13]. The MAPKs consist of three main members: the extracellular signal-regulating kinase (ERK), c-Jun N-terminal protein kinase (JNK), and p38, and are an important family of serine/threonine protein kinases implicated in inflammation [14]. MAPKs are known to be AP-1 regulators. LPS can activate MAPKs and cause AP-1 phosphorylation, eventually initiating inflammatory gene expression [8]. NF-*κ*B is a nuclear transcription factor regulating the transcription of various genes involved in inflammation [15]. Activation of NF-*κ*B promotes the expression of its downstream inflammatory cytokines such as MCP-1, IL-6, IL-1, and TNF-α [16,17,18], and eventually induces an inflammatory response [19]. Thus, the AP-1, MAPK, and NF-κB pathways are therapeutic and preventive targets in inflammatory diseases.

**Figure 1:**
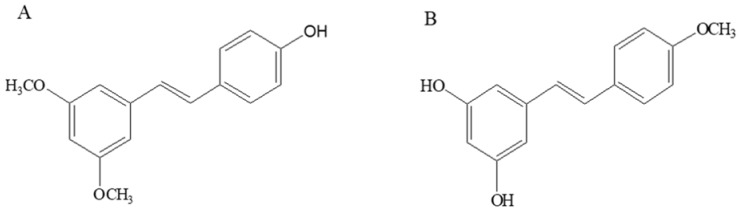
Chemical structure of (**A**) pterostilbene (Pte) and (**B**) 4′-methoxyresveratrol (4MR).

Pterostilbene (trans-3,5-dimethoxy-4-hydroxystilbene, Pte) (Figure 1A), a dimethylated analog of resveratrol, is rich in small berries such as grapes and blueberries [20]. Pte shows higher lipophilicity than resveratrol due to its methoxy substitution and therefore has a higher oral bioavailability compared with resveratrol [21]. Pte has been reported to have anti-inflammatory activity by others [22,23]; 4′-methoxyresveratrol (3,5-dihydroxy-4′-methoxystilbene, 4MR) (Figure 1B) is a monomethylated analogue of resveratrol found in the Dipterocarpaceae and Gnetaceae, the anti-inflammatory activity of which has never been studied so far. 

In the present study, we measured the anti-inflammatory activities of Pte and 4MR using a RAW264.7 macrophage model of inflammation. The concentrations of NO in the culture medium of Pte- and 4MR-treated inflammatory cells were measured. The mRNA expression levels of inducible nitric oxide synthase (iNOS) iNOS as well as the proinflammatory cytokines MCP-1, IL-6, IL-1β, and TNF-α were evaluated by quantitative real-time PCR (qPCR) after Pte and 4MR treatment. In addition, the effects of Pte and 4MR on MAPK, AP-1, and NF-κB pathways were examined to explore their mechanism of action in LPS-induced macrophages.

## 2. Results

### 2.1. Effects of Pte and 4MR on Cell Viability

**Figure 2:**
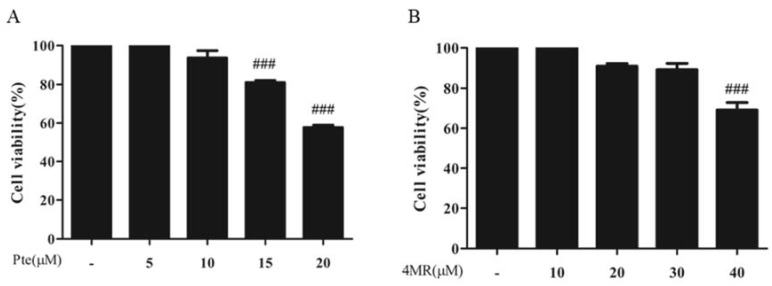
Crystal violet assay showed the effect of (**A**) Pte and (**B**) 4MR on cell viability. Cells were treated with 0–20 µM of Pte and 0–40 µM 4MR of for 24 h. The data were representative of the three independent experiments and presented as the mean ± SEM. ### *p* < 0.001 compared to control.

The effects of Pte and 4MR on cell viability was examined in RAW264.7 cells. As shown in Figure 2, cells treated with Pte at 0–10 µM or 4MR at 0–30 µM for 24 h showed no significant cytotoxicity (*p* ≥ 0.05). Thus, 5 µM of Pte and 4MR was used for the subsequent experiments to exclude the possibility that the inhibitory effect of Pte and 4MR on LPS-induced inflammation was a result of cytotoxicity caused by cell viability reduction.

### 2.2. Pte and 4MR Inhibited LPS-Induced NO Production by Attenuating iNOS Expression in RAW 264.7 Macrophages 

**Figure 3:**
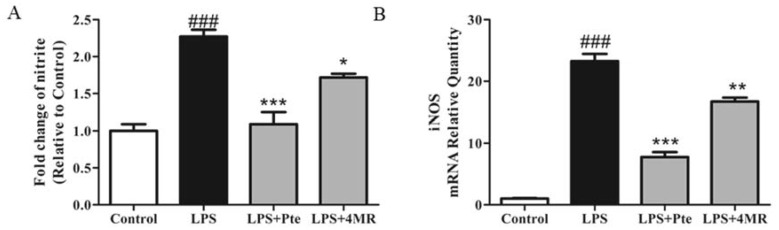
Effect of Pte and 4MR on lipopolysaccharide (LPS)-induced (**A**) nitrite production and (**B**) iNOS mRNA expression in RAW264.7 cells. Cells were cotreated with both LPS (0.1 µg/mL) and Pte (5 µM) or 4MR (5 µM) for 24 h. The NO levels in the culture medium were determined using the nitrate reductase method. The mRNA expression levels were evaluated by qRT-PCR and normalized to the 18S levels. The data were representative of the three independent experiments and presented as the mean ± SEM. ### *p* < 0.001 compared to control; *p < 0.05, ** *p* < 0.01, and *** *p* < 0.001 compared to LPS alone.

Nitric oxide (NO) is a proinflammatory mediator that can cause a systemic inflammatory response [24]. The concentration of nitrite in the culture medium was determined using the Griess reaction to evaluate the effect of Pte and 4MR on NO production in LPS-stimulated RAW264.7 macrophages. As shown in Figure 3A, both Pte and 4MR treatment suppressed LPS-induced production of NO significantly, and Pte showed a two times more potent inhibitory effect on NO production than 4MR.

Inducible nitric oxide synthase (iNOS) was the major rate-limiting enzyme that regulated NO synthesis [24]. The expression of iNOS was measured at mRNA levels by qRT-PCR to investigate whether Pte and 4MR inhibited NO production via modulation of iNOS mRNA expression. As shown in Figure 3B, Pte and 4MR inhibited LPS-induced gene expression of iNOS in RAW264.7 macrophages, implying that Pte and 4MR inhibited the LPS-induced NO production through suppressing the activity of iNOS.

### 2.3. Pte and 4MR Inhibited LPS-induced Proinflammatory Cytokine Gene Expression

**Figure 4:**
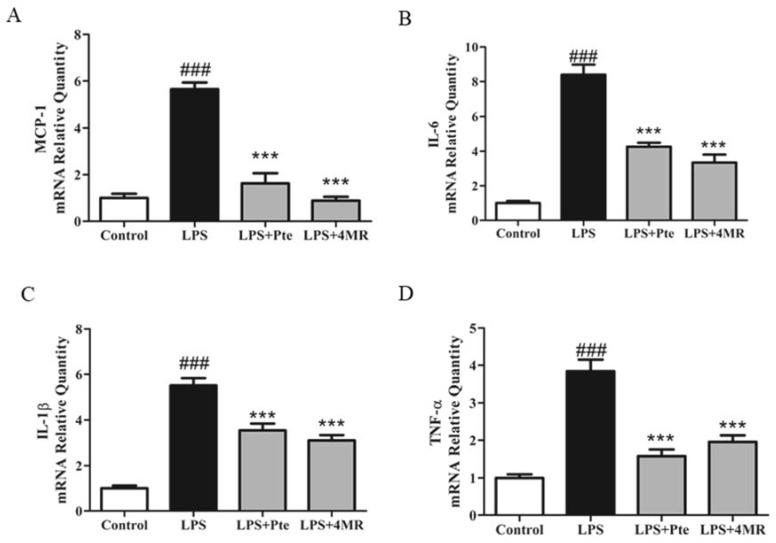
Effect of Pte and 4MR on LPS-induced proinflammatory cytokines expression in RAW264.7 cells. Cells were cotreated with both LPS (0.1 µg/mL) and Pte (5 µM) or 4MR (5 µM) for 24 h. The mRNA expression levels of (**A**) MCP-1 (**B**) IL-6 (**C**) IL-1β (**D**) TNF-α were evaluated by qRT-PCR and normalized to the 18S levels. The data were representatives of three independent experiments and presented as the mean±SEM. ### *p* < 0.001 compared to control; *** *p* < 0.001 compared to LPS alone.

As proinflammatory cytokines play a pivotal role in regulating immune responses in various inflammatory conditions, we next examined the mRNA expressions of proinflammatory cytokines including MCP-1, IL-6, IL-1β, and TNF-α to determine whether Pte and 4MR regulated their expression, using qRT-PCR. As shown in Figure 4, both Pte and 4MR treatment inhibited LPS-induced MCP-1, IL-6, IL-1β, and TNF-α mRNA expression in RAW 264.7 macrophages.

### 2.4. Pte and 4MR Inhibited LPS-Induced NF-κB Signaling Pathway in RAW 264.7 Macrophages

**Figure 5:**
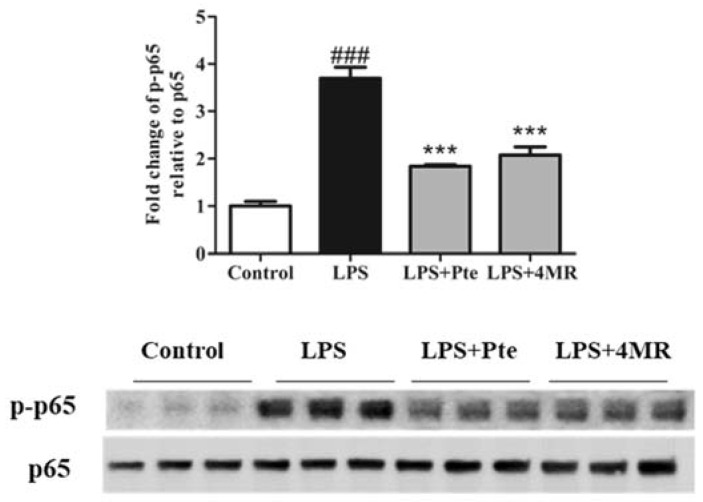
Effect of Pte and 4MR on LPS-induced p-p65 protein expression in RAW264.7 cells. Cells were cotreated with both LPS (0.1 µg/mL) and Pte (5 µM) or 4MR (5 µM) for 30 min. The protein expression levels were measured by Western blot. The data were representatives of three independent experiments and presented as the mean ± SEM. ### *p* < 0.001 compared to control; *** *p* < 0.001 compared to LPS alone.

The transcription factor nuclear factor-kappa B (NF-κB) plays an important role in regulating proinflammatory cytokines. Activated NF-κB translocated to the nucleus to bind target DNA, which in turn regulated the expression of various of inflammatory cytokines [25]. Therefore, we examined whether Pte and 4MR has a suppressive effect on phosphorylation of p65 in LPS-induced macrophages by Western blotting. Figure 5 showed that both Pte and 4MR significantly attenuated p65 phosphorylation, demonstrating that Pte and 4MR inhibited proinflammatory cytokine gene expression by suppressing the NF-κB pathway.

### 2.5. Pte and 4MR Inhibited LPS-Induced MAPK and AP-1 Pathways in RAW 264.7 Macrophages

**Figure 6:**
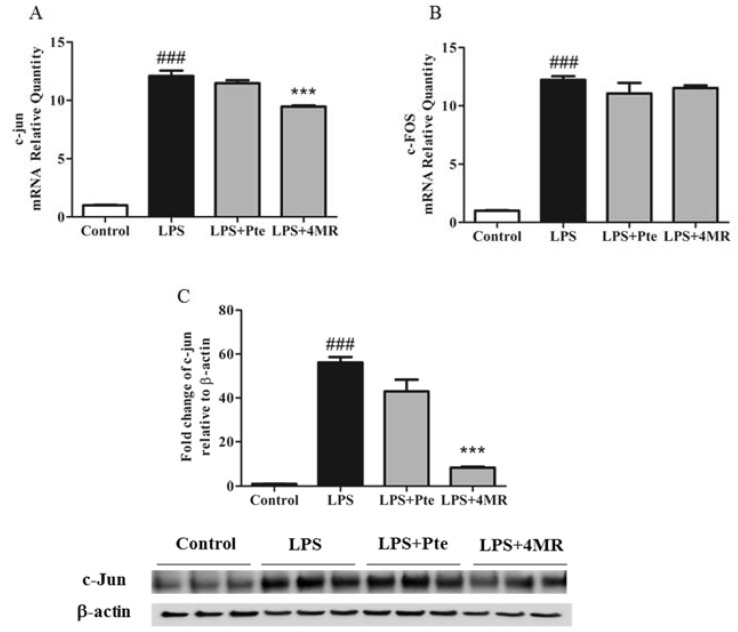
Effect of Pte and 4MR on LPS-induced c-Jun and c-Fos mRNA expression and c-Jun protein expression in RAW264.7 cells. (**A**,**B**) Cells were cotreated with both LPS (0.1 µg/mL) and Pte (5 µM) or 4MR (5 µM) for 45 min. The mRNA expression levels were evaluated by qRT-PCR and normalized to the 18S levels. (**C**) The protein expression levels were measured by Western blot (c-Jun/b-actin). The data were representatives of three independent experiments and presented as the mean ± SEM. ### *p* < 0.001 compared to control; *** *p* < 0.001 compared to LPS alone.

AP-1 is an important inflammation-related transcription factor which is involved in regulating chronic inflammatory diseases, including multiorgan disease and rheumatoid arthritis [26]. Therefore, we investigated the inhibitory effect of Pte and 4MR on AP-1 components (c-Fos and c-Jun). Our results indicated that 4MR treatment attenuated LPS-induced c-Jun at the mRNA and protein levels, but not for c-Fos, indicating inhibition of the AP-1 pathway, while Pte had no effect on the expression of LPS-induced c-Jun and c-Fos (Figure 6). Thus, 4MR but not Pte inhibited the LPS-induced AP-1 pathway in RAW 264.7 macrophages.

**Figure 7:**
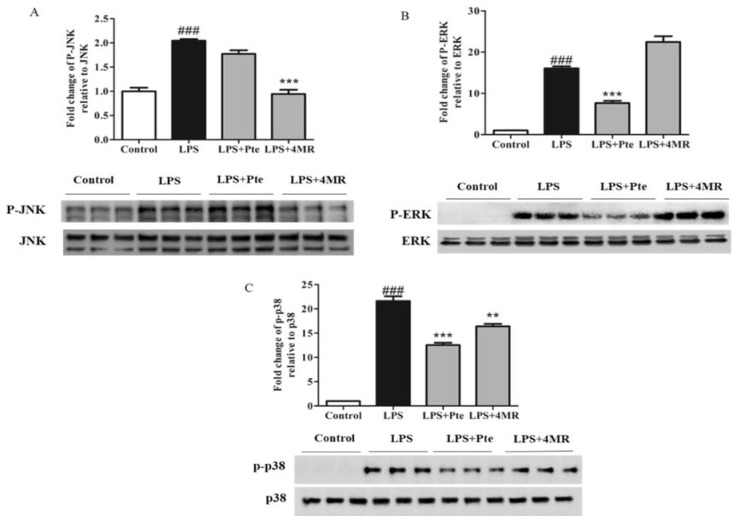
Effect of Pte and 4MR on LPS-induced (**A**) p-JNK, (**B**) p-ERK, and (**C**) p-p38 protein expression in RAW264.7 cells. The protein expression levels were measured by Western blot. Cells were cotreated with both LPS (0.1 µg/mL) and Pte (5 µM) or 4MR (5 µM) for 30 min. The data were representatives of three independent experiments and were presented as the mean ± SEM. ### *p* < 0.001 compared to control; ** *p* < 0.01, *** *p* < 0.001 compared to LPS alone.

MAPKs, including JNK, ERK, and p38, are important enzymes involved in inflammation-related signaling pathways. Importantly, MAPK signaling pathways are known to regulate AP-1 activity [27]. Thus, we next studied the effect of Pte and 4MR on the LPS-induced MAPK pathway in RAW 264.7 macrophages. As shown in Figure 7, we observed that 4MR had an inhibitory effect on JNK and p38 activation, but not on ERK, while Pte had an inhibitory effect on ERK and p38 activation, but not on JNK. These results indicated that 4MR inhibited the LPS-induced MAPK and AP-1 activation, while Pte only inhibited the LPS-induced MAPK activation in RAW 264.7 macrophages.

## 3. Discussion

Pte and 4MR are stilbenes belonging to a family of polyphenols. They are both resveratrol analogues, which are supposed to have resveratrol-like activities. In the present study, we compared the anti-inflammatory effects of Pte and 4MR in LPS-stimulated RAW264.7 cells. Both of them exhibited inhibitory potential on inflammation, but acted via different molecular mechanisms. Generally, both 4MR and Pte inhibited production of NO and gene expression of related enzymes. Both stilbenes significantly suppressed the mRNA expression of proinflammatory cytokines (MCP-1, IL-6, IL-1β, and TNF-α). Moreover, Pte inhibited inflammation mainly by blocking the MAPK-mediated NF-κB pathway, while the anti-inflammatory effect of 4MR was via the crosstalk between JNK and AP-1, as well as MAPK and NF-κB.

Attraction and activation of macrophages is regulated by numerous chemokines and cytokines, which are predominantly regulated by certain transcription factors. NF-κB is a primary transcription factor extensively involved in inflammation in various cells, activated via MAPK signaling cascades. NF-κB is sequestered by the inhibitor of κB combined with RelA (p65), and p65 can be released upon phosphorylation when activated. The phosphorylated p65 translocates to the nucleus to bind target DNA, which in turn promotes the transcription of proinflammatory genes [28,29]. AP-1 is also a crucial transcription factor regulating the inflammatory response. Activated AP-1 can bind to the AP-1 locus on the promoter of proinflammation genes to regulate their transcription, thereby participating in stress, inflammation, and other pathological processes. c-Jun is a key component of AP-1 and has been indicated in vitro as an integration point for numerous signals [30]. LPS is a well-known inducer of inflammation in macrophages, cancer cells, and epithelial cells, although the inflammatory gene expression varies with cell type. Increasing evidence showed that NF-κB and AP-1 are necessary for LPS-induced inflammation [31]. Furthermore, p65 and c-Jun are commonly used as markers of the activation of the AP-1 and NF-κB pathways. Therefore, the expression and phosphorylation of p65 and c-Jun are measured in the present study, to reflect the regulation of the NF-κB and AP-1 pathways caused by the two polyphenols.

Pte is known to exert anti-inflammatory properties in various types of cells. Qureshi et al. reported that Pte suppressed LPS-stimulated inflammation in RAW264.6 macrophages and peritoneal macrophages from C57BL/6and BALB/c mice [32]. Inflammatory iNOS and COX-2 gene expression as well as secretion of TNF-α, IFN-γ, and IL-1β were reduced by Pte. It is suggested that the anti-inflammatory effect was mediated by inhibiting NFκB activation through interfering with PI3K/Akt and MAPK [22]. Similarly, with macrophages, Pte also revealed anti-inflammatory activity in HT-29 colon cells treated with a combination of TNF-α, IFN-γ, and LPS [23]. Consistent with the findings, we found that Pte inhibited the production of NO via suppressing iNOS mRNA expression. Pte also inhibited LPS-induced gene expression of proinflammatory cytokines such as MCP-1, IL-6, IL-1β, and TNF-α, as well as other anti-inflammatory compounds such as resveratrol, quercetin, and plumbagin [10,33,34]. The molecular mechanism of Pte interacting with NF-κB was well illustrated by many previous studies. However, the function of AP-1 was neglected by researchers. In this study, we measured the AP-1 expression after Pte treatment on both the transcriptional and post-transcriptional levels. The results showed that Pte had no significant alteration on AP-1 expression in macrophages. Moreover, we investigated the influence of Pte on JNK, ERK, and p38 activation in LPS-induced RAW 264.7 macrophages. Our results indicated that Pte suppressed LPS-induced ERK and p38 phosphorylation, but not JNK. In addition, we found an inhibitory effect of Pte on LPS-induced p65 phosphorylation. Thus, Pte reduced the inflammatory response in LPS-induced RAW264.7 cells through blocking the activation of the MAPK and NF-κB pathways.

Although the anti-inflammatory effect of Pte is extensively studied, there are currently no reports on 4MR alleviating inflammation. Here, we found for the first time that 4MR, like Pte, suppressed LPS-induced inflammation in LPS-stimulated RAW264.7 cells, as evidenced by the reduced production of NO; decreased expression of proinflammatory cytokines including MCP-1, IL-6, IL-1β, and TNF-α; and inhibited phosphorylation of NF-κB, as well as p38 and JNK pathway. Moreover, unlike Pte, 4MR also inhibited the AP-1 pathway in LPS-induced macrophages, suggesting a more broad action on nuclear transcription factors.

As we reported, both 4MR and Pte have strong anti-inflammatory activities, but through different mechanisms of action. The difference might rely on the structural difference, as two hydrogen atoms are replaced by methyl on the 3- and 5-positions of the benzene ring in Pte, and only one hydrogen atom is replaced by methyl on the 4-position in 4MR when compared with resveratrol. 

## 4. Materials and Methods 

### 4.1. Materials and Reagents

Pte (≥99%, purity) and 4MR (≥98%, purity) were purchased from Great Forest Biomedical (Hangzhou, China). LPS (Escherichia coli 0111: B4), dimethylsulfoxide (DMSO), and crystal violet were purchased from Sigma-Aldrich (St. Louis, MO, USA). TRIzol reagent was purchased from Takara (Otsu, Shiga, Japan). Nitric oxide (NO) assay kit and bicinchoninic acid (BCA) protein assay kit were obtained from Nanjing Jiancheng Bioengineering Institute (Nanjing, China). Primary antibodies against ERK1/2, phospho-ERK1/2, JNK, phospho-JNK, c-Jun, phospho-c-Jun, p38, phospho-p38, p65, and phospho-p65 were obtained from Cell Signaling Technology (Boston, MA, USA). Anti-β-actin antibody was obtained from AbCam (Cambridge, Britain).

### 4.2. Cell Culture

RAW264.7 cells were purchased from Chinese Academy of Sciences (Shanghai, China). Cells were cultured in high-glucose DMEM (GIBCO, Grand Island, NY, USA) supplemented with 10% FBS (Hyclone, Logan, UT, USA) and 1% penicillin and streptomycin (GIBCO, Grand Island, NY, USA) at 37 °C under a humidified atmosphere with 5% CO_2_.

### 4.3. Crystal Violet Assay for Cell Viability

The effect of Pte and 4MR on cell viability was determined by crystal violet assay. In brief, RAW264.7 cells were seeded in a 96-well plate at a density of 2 × 10^4^ cells for 12 h and further treated with Pte (0–20 µM) and 4MR (0–40 µM) for another 24 h. After aspirating the medium, 0.5% crystal violet was added to each well. Then, the plate was washed in a stream of tap water and dried in air. The amount of dye taken up by the monolayer was quantitated with a plate reader (Synergy^TM^ Mx, BioTek, Winooski, VT, USA) at 570 nm. 

### 4.4. Measurement of NO

Cells were seeded in a 24-well plate at a density of 2 × 10^5^ cells per well. After 24 h, the cells were incubated with 5 µM of Pte and 4MR in the presence of LPS (0.1 µg/mL) for 24 h. To determine the concentration of nitrite in the culture media, Griess reagent (1% sulfanilamide, 0.1% naphthylethylenediamine dihydrochloride and 5% phosphoric acid) was added to 50 μL of supernatant at each treatment condition and the absorbance at 550 nm was measured with a microplate reader.

### 4.5. RNA Extraction and Quantitative PCR Analysis

Raw264.7 cells were seeded on a 6-well plate at 2 × 10^6^ cells per well and further treated with Pte and 4MR with or without LPS for 24 h. Total RNA was isolated using TRIzol reagent. The concentration of total RNA was measured by a spectrophotometer (Nanodrop, Invitrogen, Wilmington, DE, USA). Complementary DNA (cDNA) was synthesized with the PrimeScript^TM^ reagent Kit containing gDNA Eraser (Takara, Otsu, Shiga, Japan). Gene expression was determined with real-time qPCR using FastStart Essential DNA Green Master Kit (Roche, Basel, Switzerland) as previously described. Primer sequences are listed in Table 1. The target genes expression was normalized to 18S. Data were presented as the fold change of each sample group relative to the control group.

### 4.6. Western Blot Analysis

Whole cell lysates of treated RAW264.7 cells were prepared with RIPA lysis buffer containing protease and phosphatase inhibitors. After incubating on ice for 15 min, cell extracts were centrifuged for 10 min at 12,000 g at 4 °C to obtain cell protein. Protein (20 μg) was separated by 10% SDS-PAGE gel (Bio-Rad, San Diego, CA, USA) and transferred to polyvinylidene difluoride (PVDF) membranes with Trans-Blot^®^ Turbo^TM^ (Bio-Rad, San Diego, CA, USA). After blocking for 1 h at room temperature, the membrane was incubated with primary antibodies overnight at 4 °C. The PVDF membranes were washed three times in PBS-T (5 min each) prior to incubation with horseradish peroxidase (HRP) conjugated secondary antibody for 30 min at room temperature. This was followed by three 5 min washes with PBS-T. The membranes were reacted with enhanced chemiluminescence (ECL) reagents for 1–3 min and exposed by ChemiDoc^®^ MP Image Lab (Biorad, San Diego, CA, USA). The band densities were determined by an image Lab Software (Biorad, San Diego, CA, USA).

### 4.7. Statistical Analysis

All results were expressed as means ± SEM. Statistical analysis was performed with one-way ANOVA followed by Tukey’s multiple comparison test using Prism 5.0, GraphPad Software (5.0, San Diego, CA, USA). Results with *p*-value < 0.05 were considered as statistically significant.

## 5. Conclusions

In the present study, both Pte and 4MR exhibited inhibitory potential on inflammation in LPS-stimulated RAW264.7 cells but acted via different molecular mechanisms. Pte showed anti-inflammatory activity by blocking MAPK and NF-κB signaling pathway, while 4MR through suppression of MAPK, AP-1, and NF-κB signaling pathway. These two chemicals might serve to prevent inflammation-related disease.

## Figures and Tables

**Table 1:** Primers used for qPCR analysis.

Gene	Forward Primer	Reverse Primer
MCP-1	AGCTCTTTCCTCCACCA	CTACAGCTTCTTTGGGACACCT
IL-6	AGCCAGAGTCCTTCAGAGAGAT	GCACTAGGTTTGCCG AGTAGAT
IL-1β	GCAACTGTTCCTGAACTCAACT	ATCTTTTGGGGTCCGTCAACT
TNF-α	CACCACGCTCTTCTGTCTACTG	CTTGAGATCCATCGCGTTG
iNOS	GGCAGCCTGTGAGACCTTTG	GCATTGGAAGTGAAGCGTTTC
c-Jun	CCTTCTACGACGATGCCCTC	AGAAGGTCCGAGTTCTTGGC
c-Fos	CGGGTTTCAACGCCGACTA	TGGCACTAGAGACGGACAGAT
18S	GTAACCCGTTGAACCCCATT	CCATCCAATCGGTAGTAGCG
